# Fragments and hot spots in drug discovery

**DOI:** 10.18632/oncotarget.4968

**Published:** 2015-07-21

**Authors:** Sandor Vajda, Adrian Whitty, Dima Kozakov

**Affiliations:** Department of Biomedical Engineering and Department of Chemistry, Boston University, Boston, MA, USA

**Keywords:** ligand design, fragment conservation, druggability

Drug discovery requires finding a “plug” that fits extremely well and with high specificity into a functional site on a protein target. This difficult task often requires screening of very large compound libraries, and on occasion screening finds no useful compounds at all. For many years, a failed screening effort left unanswered whether this outcome resulted from the absence of suitable compounds in the particular screening library used, or instead indicated that the target contained no “druggable” site, and was therefore intractable for small molecule drug discovery. This dilemma was partly resolved by the advent of fragment-based drug discovery (FBDD) [1], a method that exploits the fact that it is easier to find a very small, simple compound that complements just a small region within a binding site than it is to find a larger, more complex molecule that matches the entire site [2]. In FBDD the target of interest is screened against a library of low molecular weight (typically 150-250 Da) compounds called “fragments”. Compared to conventional high throughput screening, fragment screens give much higher hit rates, and typically require testing of only 1000-2000 compounds to establish the druggability of the target and to identify initial hits [1].

Due to their small size, fragments bind with very low affinity, a *K_D_* of 1 mM being typical, and so require extensive elaboration to achieve the high affinity and other properties required for a useful lead compound. Selecting which fragment hits provide the best starting points for drug discovery is thus a critical decision. The concept of Ligand Efficiency [3] has provided an important quantitative tool for assessing fragment hit quality. However, until recently it has been unclear how to tell which fragment hits additionally have a binding mode that is sufficiently robust to serve as a basis for growing into a larger, higher affinity ligand. Moreover, although it has been recognized for some time that the hit rate for a given target in a fragment screen gives a good indication of the potential to ultimately obtain a high affinity, druglike ligand, the theoretical basis for this observation has been unclear.

Recent work has shown that considering a binding site in terms of the number, strength and spatial arrangement of binding energy “hot spots” provides answers to these questions. Different regions of a protein surface have different abilities to interact with ligands, depending on local concavity and physicochemical composition. Regions that can contribute large amounts of binding energy are called hot spots [4], and can be identified experimentally through alanine scanning mutagenesis or by their tendency to bind organic solvents or fragments, or by computational methods. Our own contributions have used the computational method FTMap [5], in which small organic probes are positioned on a dense grid around the surface of the protein, and those probes that interact favorably are identified, subjected to energy minimization, and clustered. This process is repeated using multiple probe types, the results from which are superimposed. Sites that bind a large number of probe clusters coincide with binding energy hot spots. The energetic importance of hot spots on a given protein is determined by the number of probe clusters that overlap at each site.

In recent years we have established that the hot spots identified by FTMap agree with those identified by experimental fragment screening, with the results of alanine scanning mutagenesis studies, and with the binding site locations of known inhibitors. Recently we have shown that hot spot analysis can be used to evaluate whether a protein is druggable by small molecule inhibitors [6]. As might be expected, a protein without a strong hot spot cannot bind any ligand with high affinity. We have also shown that the top-ranked hot spot dominates ligand binding, such that only fragments that achieve good spatial overlap at this main hot spot are very likely to maintain their binding mode as the fragment is extended to form a larger, stronger-binding ligand [7]. A consequence is that a target with a main hot spot that is too large and diffuse to be encompassed by a fragment-sized ligand may be a poor choice for FBDD, even though it is potentially druggable using other methods. The locations of other nearby hot spots provide important guidance as to which directions to grow the fragment to increase affinity.

Overall, this recent work shows that the strengths and arrangement of hot spots can be used to establish whether a target is druggable, whether FBDD is an appropriate approach for lead identification, to evaluate fragment hits to identify those with the best potential for advancement, and to suggest in which directions fragments should be extended for increased affinity. For targets that are not druggable using conventional druglike compounds, hot spot analysis using FTMap provides insight into whether they might be addressed using larger, non-canonical drug chemotypes such as macrocycles [6]. Importantly, this information is obtained using the X-ray crystal structure of the unbound protein, so is useful for novel or highly challenging targets for which no ligands are known. The hot spots of any protein can be easily determined using the free FTMap server (http://ftmap.bu.edu/) [5].
